# The Impact of Environmental Uncertainty on Corporate Innovation: Empirical Evidence from an Emerging Economy

**DOI:** 10.3390/ijerph19010334

**Published:** 2021-12-29

**Authors:** Jinyong Chen, Weijia Shu, Xiaochi Wang, Muhammad Safdar Sial, Mariana Sehleanu, Daniel Badulescu

**Affiliations:** 1 Business School, Hubei University, Wuhan 430000, China; junkswv@163.com (W.S.); wxchubukj@163.com (X.W.); 2 Department of Management Sciences, COMSATS University Islamabad (C.U.I.), Islamabad 44000, Pakistan; safdarsial@comsats.edu.pk; 3 Department of Economics and Business, Faculty of Economic Sciences, University of Oradea, 410087 Oradea, Romania; mavancea@uoradea.ro (M.S.); dbadulescu@uoradea.ro (D.B.)

**Keywords:** environmental uncertainty, information transparency, enterprise technology innovation, government subsidies

## Abstract

The paper analyzes the effect of environmental uncertainty on corporate technological innovation from the perspective of an innovation value chain under the institutional background of China. This paper not only discusses the intermediary effect of agency problems on environmental uncertainty and corporate technological innovation but also deeply explores the influence of information transparency, government subsidies, and other mechanisms to alleviate agency problems on environmental uncertainty and corporate technological innovation. We use the data of listed companies in China from 2008 to 2019 as the research sample, and the results show that, in general, environmental uncertainty has a negative effect on both input and output of technological innovation, and the negative effect can last for two years. Further research shows that the agency problem has an intermediary effect on the environmental uncertainty and corporate technology innovation, and the environmental uncertainty aggravates the agency problem, which hinders the input and output of corporate technology innovation. As an important mechanism to alleviate the agency problems, information transparency and government subsidies can effectively alleviate the agency conflict, thus reducing the inhibition of environmental uncertainty on the input and output of technological innovation. Our findings contribute to the discussion of driving factors for technological innovation in the context of China’s system. Our results provide useful insights into the link between environmental uncertainty and corporate innovation for economic academics and practitioners alike.

## 1. Introduction

As an emerging economy in the world, China’s economy has entered a new stage, which is different from the rapid growth period of the past 30 years, and it is in urgent need of innovation to drive high-quality economic development. As the micro-subject of economic development, the innovation activities of corporations, especially technological innovation, are not only directly related to their long-term survival and sustainable development in an increasingly fierce competitive environment but also related to the core competitiveness and competitive position of industries, regions and countries. However, corporate technological innovation faces the influence of environmental uncertainty such as trade friction, policy change, and market demand change. Environmental uncertainty is an unpredictable change, which is risky and ambiguous [1]. In addition, the agency problem is common in corporate governance in East Asian countries, which will also affect corporate technological innovation. Therefore, the paper explores the influence of environmental uncertainty on technological innovation under the institutional background of agency conflict.

The existing literature includes a significant amount of research on the motivation of corporate technological innovation. Focusing on the external influencing factors of corporate technological innovation, such as financial policy, industrial policy and industry characteristics [2,3,4], and internal influencing factors, such as corporate governance structure, managers’ shareholding ratio, executive incentive, corporation size, and nature of equity [5,6,7], a great deal of results have been achieved. However, most of the existing literature has not considered the risks faced by corporations and environmental uncertainty. The innovation investment decisions made by management are influenced by multiple factors, and any environmental uncertainty has risks, which in turn affects management’s innovation investment decisions [8]. There are two important points in the literature about the influence of environmental uncertainty on technological innovation. One is the “opportunity-oriented effect” of environmental uncertainty on corporate technological innovation [9,10], and the other is the “risk avoidance effect” [11,12].

However, the current research mainly focuses on whether the relationship between them is linear or non-linear. On the one hand, it does not deeply explore the path and mechanism of “opportunity-oriented” or “risk-avoiding” caused by environmental uncertainty to corporate technological innovation. On the other hand, the existing literature mainly focuses on the situation of developed countries in Europe and the United States. The research on the environmental uncertainty of corporate technological innovation in emerging economies in the world needs to be enriched, and its institutional background should also be considered. Based on the above analysis, the paper integrates innovation theory, information asymmetry theory and principal-agent theory. Under the institutional background of China and from the perspective of an innovation value chain, the OLS model, Poisson model and ZIP model are used to analyze the effect of environmental uncertainty on corporate technological innovation. This paper not only discusses the intermediary effect of the agency problem on environmental uncertainty and corporate technological innovation but also deeply explores the influence of information transparency, government subsidies and other mechanisms to alleviate the agency problem on environmental uncertainty and corporate technological innovation.

The contribution of the paper is reflected in three aspects: (1) On the basis of the existing literature, this paper further explores the path and mechanism of environmental uncertainty on corporate technological innovation, especially whether there is “opportunity-oriented effect” or “risk-avoiding effect” in emerging countries, which enriches the related literature on technological innovation motivation and economic consequences of environmental uncertainty; (2) combined with China’s institutional background and the prevalence of agency problems, the paper further discusses whether the environmental uncertainty affects corporate technological innovation through the agency problem, which makes up for the lack of relevant research on its influencing mechanism; (3) this paper attempts to alleviate the agency conflict from the perspective of internal information transparency and external government subsidies and integrate it into the research framework of environmental uncertainty on corporate technological innovation, so as to provide a theoretical and empirical basis for corporate technological innovation development and government departments to formulate relevant policies.

The paper is organized as follows: Section 2 provides the theoretical background and research hypothesis. Section 3 shows our methodology. Section 4 presents our results and discussion, and Section 5 shows conclusions, policy implications, limitations and future prospects.

## 2. Theoretical Background and Research Hypothesis

Innovation activity is a long-term investment activity with long cycles and high risk [13]. When the external environment situation is serious, enterprises, in order to deal with emergencies that may result from environmental uncertainty, based on preventive motivation, usually tend to choose conservative investment strategies, reduce the capital of innovative investment, and maintain a high free cash flow [8] to deal with market shocks and fierce market competition and ease the pressure of survival. When the external environment is more uncertain, the less accurate management is in judging the merits of innovative investment projects, preferring to delay innovation investment or reduce capital investment, and only when the environment stabilizes and uncertainty is manageable or disappears will companies put innovative investment projects on the agenda [14].

In the research on the motivation and economic consequences of enterprise technological innovation, many documents are often confined to a single stage of innovation activities or generally study them as a whole while ignoring the value chain of innovation activities. Innovation activities should be subdivided into different stages, and domestic scholars divided innovation activities into two stages: technology research and development and achievement transformation. The innovation capabilities of enterprises at different stages are affected by different factors, and the various stages are interrelated and interact with each other. In the technology development stage, companies will organize R&D personnel to use existing resources to develop new technologies and products by investing time and money, etc. This stage is usually manifested as a large amount of investment in R&D expenses. The greater the environmental uncertainty, the riskier the market [15]. Managers will adopt a conservative attitude in operating the enterprise, thereby reducing the enterprise’s investment in innovation.

**Hypothesis** **1a** **(H1a).** *In the technological development stage, environmental uncertainty has an inhibitory effect on technological innovation.*

In the achievement transformation stage, the investment in the research and development stage is transformed into technological achievements or new products produced, and the sales of products create income for the enterprise and bring about the improvement of the economic efficiency of the enterprise. When the uncertainty of the external environment rises, on the one hand, environmental uncertainty increases the difficulty of evaluating management’s business decisions, causing management to make decisions more cautiously and adopt a conservative or herd decision strategy in order to avoid making mistakes and damaging interests, which makes the investment level of innovation activities restricted [16]; on the other hand, increased environmental uncertainty may have a greater impact on the company and even destroy the company’s existing innovation potential, leading to short-sighted management and not accepting innovation activities that could obtain potentially high-return through taking risks to avoid risk, which hinders the technological innovation of enterprises. Accordingly, the following hypotheses are proposed:

**Hypothesis** **1b** **(H1b).** *At the stage of achievement transformation, environmental uncertainty has an inhibitory effect on technological innovation.*

Agency problems are common in modern enterprises, and information asymmetry is the main reason for increasing agency costs. Large shareholders hold a high proportion of shares and participate in the business decision-making of enterprises [17]. Based on the high cost and uncertainty of R&D projects, large shareholders may tend to avoid risks [18]. The greater the environmental uncertainty, the more difficult it is for the external supervisory authority or the media to supervise the major shareholders, which covers up the responsibility of the executives for investment failures [19], and the more difficult it is for the major shareholders’ infringement to be found, which increases the executive’s personal interest motives, and the major shareholders’ payment cost has been reduced. Major shareholders are the decision makers of major issues of the company, and technological innovation is a project that requires long-term investment and has a relatively high cost, so major shareholders may abandon R&D projects out of consideration of risks and returns [20]. Major shareholders with a high proportion of shares hold an evasive attitude towards high-risk R&D projects, and the probability of abandoning R&D increases [21]. The management mechanism and governance level of an enterprise affect the investment of innovative activities, and environmental uncertainty increases the degree of information asymmetry, creating conditions for large shareholders to encroach on the interests of small shareholders, which will exacerbate the problem of agency conflicts, resulting in a lack of the driving force of sustained high-level innovation investment by controlling shareholders. Therefore, environmental uncertainty exacerbates the emergence of agency problems, thereby further inhibiting the innovation activities of enterprises. Based on the above analysis, the following hypotheses are proposed:

**Hypothesis** **2** **(H2).** *Under the circumstance that other conditions remain unchanged, environmental uncertainty exacerbates the second type of agency problem, which has a restraining effect on enterprise technological innovation activities.*

According to the theory of information asymmetry, information asymmetry is common among investors [22]. When a company is in a high degree of environmental uncertainty, the asymmetry of information intensifies, and the decision-making of the company’s management is affected, causing R&D activities to face greater threats and weaken the enthusiasm of management for innovation [23]. The innovation activities of enterprises need financial support, so when there is a “funding gap” in the investment innovation activities of enterprises, especially small or new enterprises are vulnerable to insufficient investment caused by external factors, and there are also reasons for insufficient internal investment, which leads to an increase in the cost of enterprise innovation. It is difficult to sustain innovation activities [24]. The problem of insufficient capital for corporate innovation investment has two aspects. On the one hand, it is due to external financing constraints, and on the other hand, it is due to internal management incentives [25].

Corporate transparency reduces the sensitivity of management turnover to poor innovative output. It also increases innovative efficiency through its governance role in facilitating efficient allocation of R&D capital. These findings illuminate the unique roles and mechanisms of transparency in promoting innovation incentives and outcomes. Motivating innovation is important in many incentive problems. The optimal innovation-motivating incentive scheme exhibits substantial tolerance (or even reward), so it regulates the resistance of corporate executives to innovative activities and reduces the professional risks of executives. [26,27,28]. The increase in information transparency creates an atmosphere of tolerance for failure, thereby reducing the risks faced by managers, stimulating R&D motivation and promoting the output of results. Based on the above analysis, Hypothesis 3 is proposed:

**Hypothesis** **3** **(H3).** *As long as other conditions remain unchanged, improving information transparency can help alleviate the inhibitory effect of environmental uncertainty on enterprise technological innovation.*

The production and operation activities carried out by enterprises have their own specific market environment. When the environmental uncertainty changes, corporate investment will change with it [29]. When the market situation is good and the environmental uncertainty it faces is low, corporate management can predict and supervise technological changes and other changes in a timely and accurate manner, so as to make correct business decisions. The government provides free subsidies to provide companies with capital turnover opportunities, which help promote enterprises to carry out innovative activities. The severe market situation has increased the uncertainty in the external environment of the company, and various risks have also arisen, which has increased the degree of information asymmetry, and the management lacks sufficient information, so it is difficult to estimate the benefits and costs, and difficult to accurately assess the risk of decision-making. Internally, they will face greater operational and financial risks, and external investors cannot easily invest, which makes companies prone to greater financing constraints and increases the pressure on companies to survive.

Direct government transfer payments or indirect tax reductions provide companies with net cash flow, reduce the capital cost of R&D activities, reduce the uncertainty and risk of innovation, and help stimulate companies to invest in innovative projects [30]. Government subsidies transmit a positive signal to the outside when the uncertainty of the external environment increases, and major government financial incentives were positively influential to innovative economic performance of firms, alleviate the external moral hazard of enterprises, provide enterprises with invisible guarantees, and bring innovative resources [31].

When the external environment is uncertain, the impact of government subsidies on enterprises becomes more and more important. Government subsidies have a significant crowding-out influence on enterprises’ R&D investment behavior, and the influence is further moderated by the attributes of enterprise ownership [32,33]. The state encourages enterprises to innovate and conditionally provides government subsidies to attract enterprises to carry out technological innovation. Enterprises can take advantage of the opportunities brought about by environmental changes and the direct or indirect support of the government to increase innovation activities and gain core advantages [34]. Therefore, this paper proposes Hypothesis 4:

**Hypothesis** **4** **(H4).** *Under the circumstance that other conditions remain unchanged, government subsidies can help alleviate the inhibitory effect of environmental uncertainty on enterprise technological innovation.*

**Figure 1:**
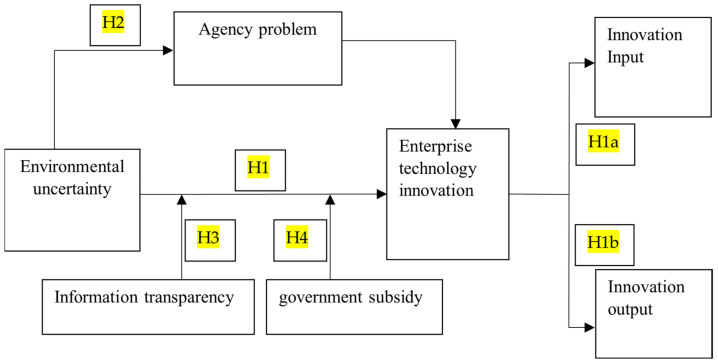
Research idea map.

The research idea of this article is shown in Figure 1.

## 3. Methodology

### 3.1. Research Samples and Data Sources

This paper obtains the financial data of the domestic listed A-share companies from 2008 to 2019 from the CSMAR database and makes full use of the data processing software Stata15 and Excel(Microsoft, Washington, WA, U.S.A) to sort and analyze. The initial research sample excluded ST listed companies, financial and insurance listed companies, samples with missing data on R&D investment, innovation output and important variables, and 10,323 samples were obtained. We winsorized the upper and lower 1% quantiles for continuous variables to reduce the deviation of extreme values from the empirical results.

### 3.2. Definition of Main Variables

#### 3.2.1. Explained Variable

This article studies innovation activities from the two stages of the innovation value chain, namely technology research and development and achievement transformation. In the technology research and development stage, the method of Hoang Luong et al. [35] is used to measure the R&D expenditures by taking the natural logarithm. In the stage of achievement transformation, the methods of Suk Bong Choi et al. [36] and Jing Chi et al. [37] are used to measure the total number of patent applications (Patents), including invention patents, exterior design, and utility models. Appendix A Table A1 shows the definitions and calculation methods of specific variables.

#### 3.2.2. Explanatory Variable

Environmental uncertainty is an explanatory variable. Environmental uncertainty has been characterized in terms of dynamism. Dynamism refers to the environmental instability that makes it difficult to predict changes and affects the volatility that a business unit faces. Typically, the volatility of industry sales and income is used to proxy dynamism [38]. In addition, the company’s operating income data from the past 5 years is used to calculate the standard deviation of abnormal sales income in the past 5 years to measure the fluctuation of its income. Then, it is adjusted in consideration of industry standards, and the industry-adjusted value is calculated as the environmental uncertainty. See Equation (1) for details.

(1) $$\mathrm{Sale}=\varphi_{0}+\varphi_{1}Year+\varepsilon$$

Among them, Sale is operating income, and Year is the annual variable

#### 3.2.3. Moderator: Information Transparency

Information transparency is reflected by accrued surplus. The larger the value, the opaquer the information. Learning from the methods of scholars Hutton, et al. [39], the accrued surplus calculated by the revised Jones model measures the severity of the whitewashing of corporate profits. The larger the Trans obtained by Equation (4), the larger the accrued surplus and the lower the information transparency. In order to facilitate the analysis, 1-Tran is used to forward the indicator.

(2) $$\begin{array}{c} {\mathrm{TA}}_{i,t}{/\mathrm{Asset}}_{i,t-1}=\alpha_{1}(1/{\mathrm{Asset}}_{i,t-1})+\alpha_{2}(\Delta REV_{i,t}/{\mathrm{Asset}}_{i,t-1})\\ \;+\alpha_{3}(\Delta PPE_{i,t}/{\mathrm{Asset}}_{i,t-1})+\varepsilon_{i,t} \end{array}$$

(3) $${\mathrm{DA}}_{i,t}={\mathrm{TA}}_{i,t}-\left\{\begin{array}{l} \alpha_{1}(1/{\mathrm{Asset}}_{i,t-1})+\alpha_{2}(\Delta REV_{i,t}-\Delta REC_{i,t})/{\mathrm{Asset}}_{i,t-1}\\ +\alpha_{3}(\Delta PPE_{i,t}/{\mathrm{Asset}}_{i,t-1}) \end{array}\right\}$$

(4) $${\mathrm{Tran}}_{i,t}=\left\{Abs(DA_{i,t-1})+Abs(DA_{i,t-2})+Abs(DA_{i,t-3})\right\}/3$$

(5) $${\mathrm{Trans}}_{i,t}=1-{\mathrm{Tran}}_{i,t}$$

Among them, TA is the total accrued surplus, Asset is the total assets of the lagging period, ΔREV is the ratio of the change in sales income to the total assets of the previous year, ΔPPE is the change in the original value of fixed assets, and ΔREC is the change in accounts receivable.

#### 3.2.4. Government Subsidy

The government subsidy in this article is the government subsidy under the non-operating income item in the notes of the financial statements of the CSMAR Database. We learned from scholars Martin, Hud et al. [40], etc., to divide this value by total assets to measure government subsidy.

#### 3.2.5. Mediator

The second type of agency problem is the mediating variable, which draws on the methods of domestic scholars and is measured by the ratio of other receivables to total assets. The ratio is expressed by agency.

**Figure 2:**
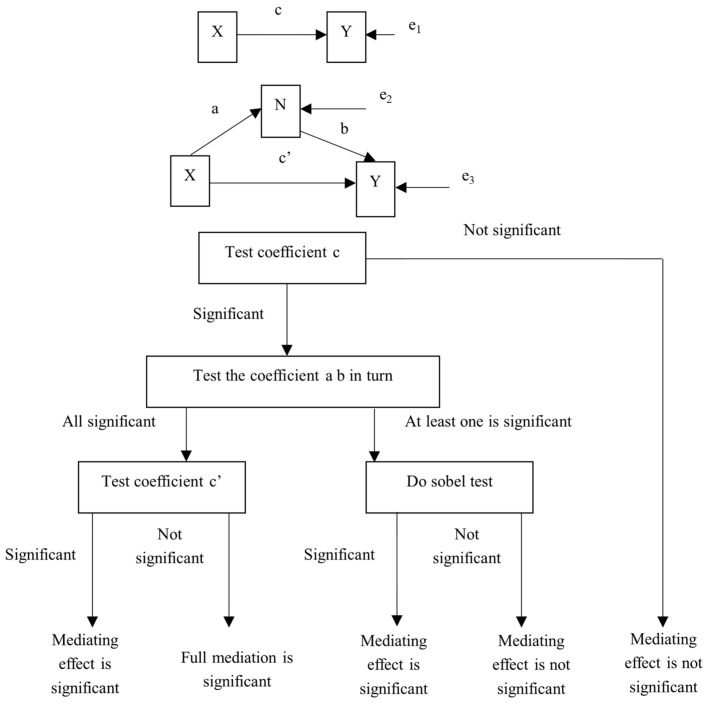
Mediating effect test procedure.

Using the mediating effect test model of Andrew Ferguson, Amanda, J., Fairchild and Mahesh, Srinivasan [41,42,43], we explored the impact of environmental uncertainty on two-stage innovation activities and the ways in which it acts, as shown in Figure 2.

(6) $$Y=\beta_{0}+\beta_{1}\mathrm{EU}+\beta_{2}\mathrm{Controls}+\beta_{3}\sum Ind+\beta_{4}\sum Year+\varepsilon$$

(7) $$Agency=\gamma_{0}+\gamma_{1}EU+\gamma_{2}\mathrm{Controls}+\gamma_{3}\sum Ind+\gamma_{4}\sum Year+\varepsilon$$

(8) $$Y=\theta_{0}+\theta_{1}Agency+\theta_{2}\mathrm{Controls}+\theta_{3}\sum Ind+\theta_{4}\sum Year+\varepsilon$$

(9) $$Y=\lambda_{0}+\lambda_{1}EU+\lambda_{2}\mathrm{Agency}+\lambda_{3}\mathrm{Controls}+\lambda_{4}\sum Ind+\lambda_{5}\sum Year+\varepsilon$$

Among them, in the inspection technology research and development stage, Y is the innovation input R&D; in the inspection achievement transformation stage, Y is the innovation output expressed by the number of patent applications (Patents) and the natural logarithm of the number of patent applications (LPatents). If *β*_1_ is significantly negative, assume that H1 is verified, *θ*_1_ is significantly positive, and *γ*_1_, *λ*_1_, and *λ*_2_ are significantly negative, then the agency problem has a significant mediating effect, and Hypothesis 2 is verified.

In order to verify the moderating effect of a moderator on environmental uncertainty and technological innovation, use the model of Mohit, Srivastava et al. [44] as reference to test innovation input; use the model of Jing, Chi et al. [37] as reference to test innovation output.

(10) $$\begin{array}{c} R\&D=\chi_{0}+\chi_{1}\mathrm{EU}+\chi_{2}M+\chi_{3}EU\times M+\chi_{4}\mathrm{ROA}+\chi_{5}\mathrm{SIZE}+\chi_{6}\mathrm{LEV}+\chi_{7}\mathrm{TOB}+\\ \;\chi_{8}\mathrm{JYYZ}+\chi_{9}\mathrm{XJBL}+\chi_{10}Cash+\chi_{11}\mathrm{Dud}+\chi_{12}\mathrm{CGB}+\chi_{13}\mathrm{Dual}+\chi_{14}\mathrm{LDBL}+\\ \;\chi_{15}\mathrm{QYCS}+\chi_{16}\sum Ind+\chi_{17}\sum Year+\varepsilon \end{array}$$

(11) $$\begin{array}{c} \mathrm{Patens}=\delta_{0}+\delta_{1}\mathrm{EU}+\delta_{2}W+\delta_{3}EU\times W+\delta_{4}\mathrm{ROA}+\delta_{5}\mathrm{SIZE}+\delta_{6}\mathrm{LEV}+\delta_{7}\mathrm{TOB}+\\ \;\delta_{8}\mathrm{JYYZ}+\delta_{9}\mathrm{XJBL}+\delta_{10}Cash+\delta_{11}\mathrm{Dud}+\delta_{12}\mathrm{CGB}+\delta_{13}\mathrm{Dual}+\delta_{14}\mathrm{LDBL}+\\ \;\delta_{15}\mathrm{QYCS}+\delta_{16}\sum Ind+\delta_{17}\sum Year+\varepsilon \end{array}$$

Model (10) draws on scholars Daniela and Coluccia, who used an OLS model [45]. For Equation (11), the dependent variable INNOVATION is the number of enterprise patent rights, so we used the Poisson model. Because the rate of sample observations of zero is 20.98%, with the phenomenon of zero-inflated, using the general Poisson model may cause the problem of biased model estimation coefficients. Therefore, this paper refers to the Zero-inflated Poisson (ZIP) model proposed by Lambert (1992), which was first proposed by Lambert (1992) and applied to the study of the damage rate of manufacturing products. Therefore, this paper uses the Zero-inflated Poisson model to conduct empirical analysis [46].

When examining the moderating effect of information transparency on environmental uncertainty and technological innovation, M, W is Trans, and introducing the interaction term between environmental uncertainty EU and information transparency Trans; when examining the moderating effect of government subsidies on environmental uncertainty and technological innovation, M, W is SUB, introducing the interaction term between EU and government subsidy SUB with environmental uncertainty.

The definitions of main variables are described in Appendix A Table A1, and we performed a Panel Unit Root Test on the variable EU to test whether the data are stable and regress the unbalanced panel data. Due to space constraints, we placed the test and regression results in Appendix A Table A2.

## 4. Results and Discussion

### 4.1. Descriptive Statistical Analysis

Table 1 shows the descriptive statistical results of each variable. It can be seen from the table that the average value of environmental uncertainty (EU) is 0.135, the minimum value is 0.013, and the maximum value is 0.961, indicating that the uncertainties faced by enterprises have differences. The median of investment in technological innovation (R&D) is 17.999, and the average is 17.950, indicating that the overall innovation investment level of listed companies is acceptable. The minimum value of innovation output, the number of patents (Patents), is 0, the average value is 75.409, and the maximum value is 4282. The gap is large, indicating that the results formed by different enterprises after investing in innovation capital are quite different and input does not necessarily bring output. As a result, the effect of inter-enterprise achievement transformation needs to be improved. The median of government subsidy (SUB) is 16.616, the maximum is 22.172, and the minimum is 0, indicating that different companies receive a large difference in the intensity of government subsidies. The minimum value of information transparency (Trans) is 0.613, the maximum value is 1.430, and the standard deviation is 0.0687. There are differences in information transparency between different companies, but the difference is not significant.

### 4.2. Correlation Analysis

Table 2 is the regression table of the correlation between the main variables. The correlation coefficients of environmental uncertainty (EU), patents (Patents) and innovation input (R&D) are −0.008 and −0.058, respectively, indicating that environmental uncertainty (EU) has a negative impact on the innovation activities of the enterprise. The correlation coefficients between government subsidies (SUB), patents (Patents) and innovation input (R&D) are 0.182 and 0.358, respectively, indicating that government subsidy (SUB) has a positive impact on the innovation activities of enterprises. The correlation coefficients between agency, patents (Patents) and innovation input (R&D) are −0.055 and −0.117, respectively, indicating that the second type of agency problem between enterprises negatively affects the technological innovation of enterprises. It can be seen from Table 2 that the correlation coefficients between the variables are all lower than 0.5. Since the VIF value of all variables is less than 10, there is no multicollinearity problem among the variables.

### 4.3. Regression Analysis

#### 4.3.1. Regression Analysis of Environmental Uncertainty and Enterprise Technological Innovation

(1) Innovation input in technology research and development stage

In order to explore the relationship between the two in depth, model (6) is used to test Hypothesis H1a to verify the relationship between environmental uncertainty and technological innovation investment. Column (1) of Table 3 shows environmental uncertainty (EU) and technological innovation regression results in the technological development stage, and the regression coefficient between EU and technological innovation input (R&D) is −0.521, which is significantly negatively correlated at the 1% confidence level, indicating that EU has an inhibitory effect on innovation input (R&D). It can be seen that the greater the environmental uncertainty, the more significant the inhibitory effect on enterprises’ investment in technological innovation, which verifies Hypothesis 1a.

Among the control variables, it can be seen from column (1) of Table 3 that the regression coefficients of enterprise size (SIZE) and profitability index (ROA) are 0.789 and 2.791, respectively, which are both significantly positive at the 1% level, indicating that the larger the scale, the better the profitability. The better innovation foundation and innovation resources companies have, the more they invest in innovation activities. The regression coefficient of investment opportunity (TOB) is positive, indicating that the more investment opportunities a company has, the more likely it is to choose an innovative project to invest in, thereby promoting the company’s input in technological innovation. The regression coefficient of the net business cycle (JYYZ) is significantly negative, which shows that the longer the net business cycle, the more unfavorable the company’s innovation investment.

(2) Innovative output at the stage of achievement transformation

In order to explore the relationship between the two in depth, model (6) is used to test Hypothesis 1b to verify the impact of environmental uncertainty on the innovation output of the enterprise in the transformation stage of the results. The results of multiple regression are shown in Table 3. Column (2) of Table 3 shows the regression results of environmental uncertainty (EU) and technological innovation in the achievement transformation stage, and innovation output (LPatents) is the natural logarithm of the number of patent applications, which is a continuous variable, using the OLS model test Hypothesis 1b. In column (2) of Table 3, the regression coefficient between EU and innovation output (LPatents) is −0.612, which is significantly negative at the 10% confidence level, indicating that EU has an inhibitory effect on innovation output (LPatents). It can be seen that the greater the environmental uncertainty, the more unfavorable the technological innovation output of the enterprise, which verifies Hypothesis 1b.

Taking into account the data characteristics of the number of patent applications in China, the explained variables of the model are converted from continuous variables to the number of patent applications to explain. As patent data have many values of 0, the Poisson model is more consistent with the number features, and multiple regression models make the regression results more robust. From column (3) of Table 3, it can be seen that the regression coefficient between environmental uncertainty (EU) and technological innovation output (Patents) is −0.344, which is a significant negative correlation, indicating that EU’s influence on innovation output (Patents) has an inhibitory effect. It can be concluded that the greater the environmental uncertainty, the more unfavorable the technological innovation output of the enterprise, which verifies Hypothesis 1.

#### 4.3.2. Regression Analysis of Environmental Uncertainty, the Second Type of Agency Problem and Enterprise Technological Innovation

In the technology research and development stage, models (6)–(9) were used to test Hypothesis 2 to verify the relationship between environmental uncertainty and technological innovation input and the mediating effect of the second type of agency problem on environmental uncertainty and technological innovation input. From column (1) of Table 4, it can be seen that the regression coefficient between environmental uncertainty EU and technological innovation input (R&D) is −0.521, which is significantly negatively correlated at the 1% confidence level, indicating that EU’s contribution to innovation input (R&D) has an inhibitory effect. Further testing the mediation effect, from column (2) of Table 4, it can be seen that the correlation coefficient between agency and innovation input (R&D) is −2.693, which is significantly negative at the 1% confidence level, indicating that the second type of agency problem is common among enterprises and affects enterprises’ input in technological innovation and inhibits innovation. From column (3) of Table 4, it can be seen that the regression coefficient between agency and the environmental uncertainty EU is 0.006, which is significantly positive at the 1% level, that is, the higher the environmental uncertainty, the more serious the enterprise’s second type of agency problem. From column (4) of Table 4, we can see that in model (9), the coefficients of agency, Environmental Uncertainty (EU) and the company’s technological innovation input (R&D) are −2.606 and −0.505, respectively, which are both significantly positive at the 1% level. Combining the regression results of the previous models, we show that the greater the environmental uncertainty, the more significant the inhibitory effect on the enterprise’s technological innovation investment. In addition, it exacerbates the second type of agency problem, which has a negative impact on enterprises’ investment in technological innovation indirectly. This verifies Hypothesis 2.

In the achievement transformation stage, we used models (6)–(9) to test Hypothesis 2 to verify the effect of environmental uncertainty on firms’ innovation output and the mediating effect of the second type of agency problem between environmental uncertainty and technological innovation output. The results of the multiple regressions are shown in Table 5. Column (1) of Table 5 shows the regression results of environmental uncertainty (EU) and technological innovation in the achievement transformation stage. Innovation output (LPatents), which is the natural logarithm of the number of patent applications, is a continuous variable, and the OLS model was used to test Hypothesis 1. In column (1) of Table 5, the regression coefficient of EU and innovation output (LPatents) is −0.612, which is significantly negative at the 10% confidence level, indicating that EU has an inhibitory effect on innovation output (LPatents). Further testing the mediating effect, in column (2) of Table 5, the correlation coefficient between agency and innovation output (LPatents) is −3.934, which is significantly negative at the 5% confidence level, indicating that the second type of agency problem is prevalent among firms, which affects their technological innovation output and plays an inhibitory role on innovation. As can be seen in column (3) of Table 5, the regression coefficient between Agency and EU is 0.006, which is significantly positive at the 1% level, that is, the higher the environmental uncertainty, the more serious the second type of agency problem of firms. In column (4) of Table 5, it can be seen that in model (9), the coefficient of agency with firms’ technological innovation output (LPatents) is −3.804, which is significantly negative, and the coefficient of environmental uncertainty (EU) with firms’ technological innovation output (LPatents) is -0.586, which is significantly negative at the 10% level. Combining the regression results of the previous models, it can be concluded that the greater the environmental uncertainty, the more unfavorable the technological innovation output of the firm, and also, it indirectly has a negative impact on the technological innovation output of the firm by exacerbating the second type of agency problem. This verifies Hypothesis 2.

Table 6 shows the regression results of the Poisson model. It can be seen from column (1) of Table 6 that the regression coefficient between environmental uncertainty (EU) and technological innovation output (Patents) is −0.344, which is significantly negative, indicating that EU’s impact on innovation output (Patents) has an inhibitory effect. To further test the mediation effect, in the column (2) of Table 6, the correlation coefficient between agency and innovation output (Patents) is −4.682, which is significantly negative, indicating that the second type of agency problem generally exists between different companies and affects the company’s output of technological innovation, inhibiting innovation. In column (3) of Table 6, the regression coefficient between agency and EU is 0.006, which is significantly positive, that is, the higher the environmental uncertainty, the more serious the second type of agency problem of the enterprise. In column (4) of Table 6, using model (9) to test, the coefficient of agency and firm’s technological innovation output (Patents) is −4.603, which is significantly negative. At the 1% confidence level, the environmental uncertainty EU and the technological innovation output (Patents) of the enterprise are significantly negative with a regression coefficient of −0.321. Combining the regression results of the previous models and using the principle of mediating the effect test, it is found that the greater the environmental uncertainty, the more unfavorable the technological innovation output of the enterprise. In addition, it also indirectly negatively affects the technological innovation output of enterprises by exacerbating the second type of agency problem. This verifies Hypothesis 2.

#### 4.3.3. Regression Analysis of Environmental Uncertainty, Information Transparency and Enterprise Technological Innovation

We used models (10) and (11) to test Hypothesis 3 to verify the moderating effect of information transparency on environmental uncertainty and technological innovation. The regression results are listed in Table 7. In the technology research and development stage, in column (1) of Table 7, at a confidence level of 1%, environmental uncertainty (EU) is significantly negatively correlated with the technological innovation input (R&D), and its regression coefficient is −2.489, indicating that EU has a restraining effect on innovation investment (R&D). After introducing the interaction terms of environmental uncertainty and information transparency, EU × Trans is significantly positively correlated at the level of 5%, and its regression coefficient is 1.982, indicating that corporate information transparency can help alleviate the inhibitory effect of the environmental uncertainty on enterprises’ investment in technological innovation.

In the stage of achievement transformation, considering the data characteristics of the number of patent applications in China, the explained variables of the model are converted from continuous variables to the number of patent applications to explain. Since patent data have many phenomena with a value of 0, the POISSON model and ZIP model test are more in line with the quantitative characteristics, and multiple regression models make the regression results more robust. Column (2) of Table 7 is the regression result of the POISSON model. It can be seen that EU and technological innovation output (Patents) are significantly negatively correlated at the 1% confidence level, and the regression coefficient is −3.225, indicating that EU has an inhibitory impact on innovation output. After introducing the interaction term of environmental uncertainty and information transparency, EU × Trans is significantly positively correlated at the 1% level, and the regression coefficient is 2.803, indicating that corporate information transparency can help alleviate the inhibitory impact of environmental uncertainty on corporate technological innovation output. Combining information transparency can alleviate the inhibitory effect of enterprises on innovation input and innovation output when facing environmental uncertainty, Hypothesis 3 in this article has been verified.

Among the control variables, it can be seen from columns (1) and (2) of Table 7 that the regression coefficients of enterprise size SIZE and profitability index ROA are both significantly positive at the 1% level, indicating that the larger the scale and the greater the profitability of the enterprise, the more it can promote the technological innovation of the enterprise. The regression coefficient of investment opportunity TOB is positive, indicating that the more investment opportunities a company has, the greater the possibility of choosing innovative projects for investment, which is conducive to promoting the technological innovation of the company. The regression coefficient of the net business cycle JYYZ is significantly negative, indicating that the longer the net business cycle, the more unfavorable to the innovation of the enterprise.

Column (3) of Table 7 is the regression result of the ZIP model. It can be seen that whether the EU × Trans (the interaction term of environmental uncertainty and information transparency) or the control variables, The results are consistent with the regression results of the OLS model and Poisson model. At the 1% confidence level, EU has a significantly negative correlation with technological innovation output (Patents), and the regression coefficient is −4.649, indicating that EU has an inhibitory effect on innovation output of patents. After introducing the interaction terms of environmental uncertainty and information transparency, EU × Trans is significantly positively correlated at the 1% level, and the regression coefficient is 4.525, indicating that corporate information transparency can help alleviate the inhibitory impact of environmental uncertainty on corporate technological innovation output. Combining information transparency can alleviate the inhibitory effect of enterprises on innovation input and innovation output when facing environmental uncertainty, Hypothesis 3 in this article has been verified.

#### 4.3.4. Regression Analysis of Environmental Uncertainty, Government Subsidies and Enterprise Technological Innovation

We used models (10) and (11) to test Hypothesis 4 to verify the moderating effect of government subsidies on environmental uncertainty and technological innovation. The regression results are listed in Table 8. In column (1) of Table 8, EU has a significant negative correlation with technological innovation input (R&D) at the 1% confidence level, and the regression coefficient is −2.650, indicating that EU has an inhibitory effect on innovation input (R&D). After introducing the interaction term between environmental uncertainty and government subsidies, EU × SUB is significantly positive at the 1% level, and the regression coefficient is 0.129, indicating that corporate government subsidies can help alleviate the inhibitory effect of EU on corporate technological innovation investment.

Table 8 shows the regression results of the POISSON model and the ZIP model. Column (2) of Table 8 is the regression result of the POISSON model. It can be seen that at the 1% confidence level, EU and technological innovation output patents are significantly negatively correlated, and the regression coefficient is −1.395, indicating that EU has an inhibitory effect on innovation output. After introducing the interaction term between environmental uncertainty and government subsidies, EU × SUB is significantly positively correlated at the 1% level with a regression coefficient of 0.064, indicating that corporate government subsidies can help alleviate EU’s inhibitory effect on corporate technological innovation output. Combining government subsidies can alleviate the inhibitory effect on innovation input and innovation output when facing environmental uncertainty, Hypothesis 4 in this paper is verified.

Among the control variables, in columns (1) and (2) of Table 8, the regression coefficients of enterprise size (SIZE) and profitability index (ROA) are both significantly positive at the 1% level, indicating that the larger the scale and the greater profitability of the enterprise, the more it can promote technological innovation. The regression coefficient of investment opportunity (TOB) is significantly positive, indicating that the more investment opportunities a company has, the more likely it is to choose an innovative project to invest, which is conducive to promoting the technological innovation of the company. The regression coefficient of the net business cycle (JYYZ) is significantly negative, indicating that the longer the net business cycle, the more adverse the effect will be on the innovation activities of the enterprise.

Column (3) of Table 8 is the regression result of the ZIP model. It can be seen that whether it is the interaction term EU × SUB between environmental uncertainty and government subsidies or the control variables, they are consistent with the regression results of the previous model. At the 1% confidence level, EU has a significant negative correlation with technological innovation output patents, and the regression coefficient is −6.46, indicating that EU has an inhibitory effect on innovation output patents. After introducing the interaction term between environmental uncertainty and government subsidies, EU × SUB is significantly positively correlated at the 1% level, with a regression coefficient of 0.365, indicating that corporate government subsidies can help alleviate EU’s inhibitory effect on corporate technological innovation output. Combining government subsidies can alleviate the inhibitory effect on innovation input and innovation output when facing environmental uncertainty, Hypothesis 4 in this paper is verified.

In addition, we have also conducted panel data regression on the main models, and the research conclusions are still consistent. See Appendix A Table A3 and Appendix A Table A4 for the specific regression results.

## 5. Conclusions, Policy Implications, Limitations and Future Prospects

### 5.1. Conclusions

Based on the existing literature research, we analyze the effects and paths of environmental uncertainty on corporate technological innovation using data from Chinese listed companies in Shanghai and Shenzhen A markets from 2008–2019. This paper not only discusses the intermediary effect of the agency problem on environmental uncertainty and corporate technological innovation but also deeply explores the influence of information transparency, government subsidies and other mechanisms to alleviate the agency problem on environmental uncertainty and corporate technological innovation, and draws the following conclusions.

First, the “risk-avoiding” effect of environmental uncertainty on corporate technology innovation is greater than the “opportunity-oriented” effect. The greater the environmental uncertainty faced by corporations, the more obvious the inhibition effect on corporate technology innovation. Specifically, the stronger the environmental uncertainty is, the less the investment in corporate innovation will be reduced, and the effect of corporations’ innovation output will be reduced in the stage of achievement transformation. Further exploration reveals that the inhibition effect of environmental uncertainty on corporate technological innovation will last for at least 2 years.

Second, the agency problem is widespread in Chinese corporations, especially the second kind of agency problem between controlling shareholders and minority shareholders. The results show that the more severe the second kind of agency problem is, the more unfavorable it is to technological innovation in listed corporations, significantly reducing corporations’ incentives to invest in sustained innovation and inhibiting innovation inputs and innovation outputs. Moreover, environmental uncertainty directly aggravates the second kind of agency problem and affects corporate technological innovation activities through agency problems, reducing corporate technological innovation inputs and innovation outputs. Further research shows that the environmental uncertainty faced by corporations can aggravate the second kind of agency problem, which will have a restraining effect on technological innovation of corporations for two years.

Third, both internal governance mechanisms (information transparency) and external governance mechanisms (government subsidies), which alleviate the agency problem, have a moderating effect on environmental uncertainty and corporate technological innovation. The improvement of information transparency and government subsidies is helpful in alleviating the inhibition effect of environmental uncertainty on corporate technological innovation inputs and outputs. Further research shows that when corporations face environmental uncertainty, they can transform the ongoing two-year negative effect of environmental uncertainty on their technological innovation by improving the transparency of corporate information.

### 5.2. Policy Implications

Based on the research conclusions, we put forward the following suggestions:

First, based on the two-stage innovation activities, in the technology R&D stage, corporations need to establish an innovation risk control mechanism oriented to environmental uncertainty, encourage corporations to optimize the allocation of corporate technological innovation resources, and maintain the stability and sustainability of R&D investment. In the achievement transformation stage, it is necessary to avoid the disconnection between R&D and transformation. Corporations should improve the output and quality of technological innovation, which will bring good revenue, thus in turn supporting technological R&D and achieving the circular development of innovation activities.

Second, corporations should continuously improve internal supervision mechanisms, improve their governance capacity and optimize corporate governance structure. Corporations are facing increasingly fierce environmental changes, while the external market changes are random and unpredictable. Corporations should improve their ability to prevent and control uncertainty, enhance their own internal governance capacity and incentive systems, and reduce moral hazards. It is necessary to give full play to the function of equity checks and balances. Corporations should improve the degree of equity checks and balances, reduce the second kind of agency problems between major shareholders and minority shareholders, and reduce the agency conflicts caused by agency problems.

Third, corporations should improve the transparency of corporate information and information disclosure mechanisms. The government should increase support for technological innovation, while strengthening the management and supervision of government subsidy funds. Both approaches are helpful to alleviate the inhibition effect of the agency problem on environmental uncertainty and corporate technological innovation. Improving the transparency of corporate information is conducive to alleviating information asymmetry. The government should give full play to the incentive role of financial subsidies, increase support for corporations eligible for subsidies, and promote the effective implementation of innovative activities. Both approaches reduce the negative impact of uncertainty and agency problems on innovation activities.

### 5.3. Limitations and Future Prospects

On the one hand, the measurement of environmental uncertainty deserves further exploration. At present, most scholars use the fluctuation of operating income to measure it, instead of measuring it from multiple dimensions that affect environmental uncertainty. On the other hand, restricted by the information disclosure and databases of Chinese corporations, technological innovation is measured only by the quantity of R&D investment and patent rights output, but quality indicators such as patent citations are not adopted. In addition, although part of the patent datum is collected manually, there are limitations due to the high number of missing values of patent data in CSMAR and other databases.

This paper explores the effect of environmental uncertainty on the technological innovation activities of corporations in two stages and its path and mechanism based on theoretical and empirical analysis research from the perspective of the innovation value chain. We provide some ideas for the study of the economic consequences caused by environmental uncertainty and also expand the research on the motivation of corporate technological innovation activities. However, the research of environmental uncertainty can be further refined in the future to explore the mechanism of its impact on technological innovation from different dimensions. On the other hand, whether there are other paths and mechanisms of environmental uncertainty affecting technological innovation can be further explored in the future.

## Figures and Tables

**Table 1:** Descriptive statistics of each variable.

Main Variable	Sample Size	Mean	Median	Minimum	Maximum	Standard Deviation
LPatents	2645	2.8497	3.1781	0.0000	8.3624	1.8491
Patents	2645	75.4091	23.0000	0.0000	4282	260.8831
R&D	10,323	17.9500	17.9999	13.7090	21.4619	1.3312
EU	10,323	0.1346	0.1005	0.0133	0.9607	0.1151
SUB	10,323	16.5139	16.6161	0.0000	22.1719	1.8988
Trans	10,323	0.9940	0.9946	0.6125	1.4299	0.0687
Agency	10,323	0.0175	0.0100	0.0002	0.2446	0.0231
ROA	10,323	0.0392	0.0346	−0.2245	0.2080	0.0475
SIZE	10,323	22.2836	22.1819	19.5409	25.8882	1.0481
LEV	10,323	0.4254	0.4205	0.0491	0.9343	0.1825
TOB	10,323	2.1097	1.7587	0.8888	8.1375	1.1429
Dual	10,323	0.7575	1.0000	0.0000	1.0000	0.4286

**Table 2:** Correlation analysis of the main variables.

Variable	Patents	R&D	EU	SUB	Trans	Agency	ROA	SIZE
Patents	1							
R&D	0.322 ***	1						
EU	−0.008	−0.058 ***	1					
SUB	0.182 ***	0.358 ***	−0.01	1				
Trans	0.005	−0.059 ***	−0.011	0.004	1			
Agency	−0.055 ***	−0.117 ***	0.054 ***	−0.011	0.015	1		
ROA	0.086 ***	0.172 ***	−0.045 ***	0.049 ***	−0.303 ***	−0.132 ***	1.000	
SIZE	0.255 ***	0.473 ***	0.055 ***	0.389 ***	−0.067 ***	−0.005	0.036 ***	1.000

Note: *** represent significance at the levels of 1%, respectively.

**Table 3:** Regression analysis of environmental uncertainty and enterprise technological innovation.

Variable	(1)	(2)	(3)
	R&D	LPatents	Patents
EU	−0.521 ***	−0.612 *	−0.344 ***
	(−6.07)	(−1.89)	(−14.70)
ROA	2.791 ***	0.332	1.035 ***
	(10.86)	(0.36)	(15.42)
SIZE	0.789 ***	0.659 ***	0.976 ***
	(58.52)	(13.72)	(333.03)
LEV	0.666 ***	1.516 ***	2.696 ***
	(5.28)	(3.22)	(57.65)
TOB	0.061 ***	0.119 ***	0.247 ***
	(5.32)	(3.08)	(94.37)
Dual	−0.070 ***	−0.310 ***	0.045 ***
	(−2.97)	(−3.82)	(8.07)
Constant	−0.970 ***	−12.975 ***	−21.093 ***
	(−3.04)	(−11.52)	(−228.64)
Industry, Year	Control		
Observation	1023	2645	2645
Adjusted R^2^	0.450	0.137	-
F	223.0	12.39	-

Note: * and *** represent significant at the 10%, and 1%, confidence levels, respectively. The t value is in parentheses.

**Table 4:** Regression analysis of environmental uncertainty, the second type of agency problem and innovation input.

Variable	(1)	(2)	(3)	(4)
	R&D	R&D	Agency	R&D
EU	−0.521 ***		0.006 ***	−0.505 ***
	(-6.07)		(3.35)	(−5.88)
Agency		−2.693 ***		−2.606 ***
		(−6.02)		(−5.83)
ROA	2.791 ***	2.771 ***	−0.010 *	2.764 ***
	(10.86)	(10.78)	(−1.84)	(10.77)
SIZE	0.789 ***	0.781 ***	−0.003 ***	0.782 ***
	(58.52)	(57.67)	(−9.22)	(57.85)
LEV	0.666 ***	0.697 ***	0.013 ***	0.701 ***
	(5.28)	(5.52)	(4.80)	(5.56)
TOB	0.061 ***	0.065 ***	0.001 ***	0.064 ***
	(5.32)	(5.63)	(4.31)	(5.57)
Dual	−0.070 ***	−0.062 ***	0.002 ***	−0.066 ***
	(-2.97)	(−2.65)	(3.03)	(−2.80)
Constant	−0.970 ***	−0.820 **	0.078 ***	−0.766 **
	(−3.04)	(−2.55)	(11.14)	(−2.39)
Industry, Year	Control			
Observation	10,323	10,323	10,323	10,323
Adjusted R^2^	0.450	0.450	0.112	0.451
F	223.0	222.9	35.22	218.8

Note: *, **, and *** represent significance at the 10%, 5%, and 1% confidence levels, respectively. The t value is in parentheses.

**Table 5:** Regression analysis of environmental uncertainty, the second type of agency problem and innovation output 1.

Variable	(1)	(2)	(3)	(4)
	LPatents	LPatents	Agency	LPatents
EU	−0.612 *		0.006 ***	−0.586 *
	(−1.89)		(3.35)	(−1.81)
Agency		−3.934 **		−3.804 **
		(−2.14)		(−2.07)
ROA	0.332	0.335	−0.010 *	0.280
	(0.36)	(0.37)	(−1.84)	(0.31)
SIZE	0.659 ***	0.646 ***	−0.003 ***	0.647 ***
	(13.72)	(13.36)	(−9.22)	(13.39)
LEV	1.516 ***	1.581 ***	0.013 ***	1.617 ***
	(3.22)	(3.35)	(4.80)	(3.42)
TOB	0.119 ***	0.122 ***	0.001 ***	0.121 ***
	(3.08)	(3.15)	(4.31)	(3.13)
Dual	−0.310 ***	−0.301 ***	0.002 ***	−0.297 ***
	(−3.82)	(−3.71)	(3.03)	(−3.65)
Constant	−12.975 ***	−12.726 ***	0.078 ***	−12.705 ***
	(−11.52)	(−11.23)	(11.14)	(−11.21)
Industry, Year	Control			
Observation	2,645	2,645	10,323	2,645
Adjusted R^2^	0.137	0.138	0.112	0.139
F	12.39	12.42	35.22	12.19

Note: *, **, and *** represent significance at the 10%, 5%, and 1% confidence levels, respectively. The t value is in parentheses.

**Table 6:** Regression analysis of environmental uncertainty, the second type of agency problem and innovation output 2.

Variable	(1)	(2)	(3)	(4)
	Patents	Patents	Agency	Patents
EU	−0.344 ***		0.006 ***	−0.321 ***
	(−14.70)		(3.35)	(−13.66)
Agency		−4.682 ***		−4.603 ***
		(−26.93)		(−26.43)
ROA	1.035 ***	0.992 ***	−0.010 *	0.992 ***
	(15.42)	(14.85)	(−1.84)	(14.79)
SIZE	0.976 ***	0.968 ***	−0.003 ***	0.969 ***
	(333.03)	(329.01)	(−9.22)	(328.79)
LEV	2.696 ***	2.723 ***	0.013 ***	2.757 ***
	(57.65)	(58.54)	(4.80)	(59.11)
TOB	0.247 ***	0.248 ***	0.001 ***	0.246 ***
	(94.37)	(94.81)	(4.31)	(93.90)
Dual	0.045 ***	0.053 ***	0.002 ***	0.051 ***
	(8.07)	(9.61)	(3.03)	(9.22)
Constant	−21.093 ***	−20.892 ***	0.078 ***	−20.881 ***
	(−228.64)	(−225.78)	(11.14)	(−225.60)
Industry, Year	Control			
Observation	2645	2645	10,323	2645

Note: * and *** represent significance at the 10%, 5%, and 1% confidence levels, respectively. The t value is in parentheses.

**Table 7:** The moderating effect of information transparency on environmental uncertainty and enterprise technological innovation.

Variable	(1)	(2)	(3)
	R&D	Patents	Patents
EU	−2.489 ***	−3.225 ***	−4.649 ***
	(−2.67)	(−10.17)	(−15.05)
Trans	0.550 **	2.684 ***	2.665 ***
	(2.45)	(43.38)	(44.01)
EU × Trans	1.982 **	2.803 ***	4.525 ***
	(2.12)	(8.83)	(14.59)
ROA	3.368 ***	4.852 ***	5.860 ***
	(13.62)	(77.33)	(89.87)
SIZE	0.794 ***	0.993 ***	0.962 ***
	(58.86)	(336.52)	(320.35)
LEV	0.657 ***	2.504 ***	1.570 ***
	(5.22)	(53.37)	(33.74)
TOB	0.061 ***	0.246 ***	0.220 ***
	(5.31)	(93.33)	(84.09)
Dual	−0.070 ***	0.050 ***	0.114 ***
	(−2.99)	(9.06)	(20.72)
Constant	−1.618 ***	−23.954 ***	−23.009 ***
	(−4.04)	(−208.74)	(−200.87)
Industry, Year	Control		
Observation	10,323	2645	2645
Adjusted R^2^	0.451	-	-
F	218.8	-	-

Note: ** and *** represent significance at the 5% and 10%, confidence levels, respectively. The t value is in parentheses.

**Table 8:** The moderating effect of government subsidies on environmental uncertainty and enterprise technological innovation.

Variable	(1)	(2)	(3)
	RD	Patents	Patents
EU	−2.650 ***	−1.395 ***	−6.460 ***
	(−3.24)	(−4.59)	(−18.95)
SUB	0.083 ***	0.270 ***	0.212 ***
	(9.96)	(80.83)	(62.95)
EU × SUB	0.129 ***	0.064 ***	0.365 ***
	(2.62)	(3.70)	(18.82)
ROA	2.947 ***	3.812 ***	4.480 ***
	(12.50)	(59.99)	(69.26)
SIZE	0.702 ***	0.725 ***	0.702 ***
	(49.51)	(197.69)	(186.77)
LEV	0.607 ***	1.887 ***	1.144 ***
	(4.88)	(41.18)	(25.57)
TOB	0.055 ***	0.212 ***	0.189 ***
	(4.87)	(82.56)	(73.37)
Dual	−0.062 ***	0.037 ***	0.130 ***
	(−2.68)	(6.69)	(23.50)
Constant	−0.415	−19.985 ***	−18.276 ***
	(−1.25)	(−198.85)	(−176.29)
Industry, Year	Control		
Observation	10,323	2,645	2,645
Adjusted R^2^	0.465	-	-
F	231.4	-	-

Note: *** represent significant at the 1% confidence levels, respectively. The t value is in parentheses.

## Data Availability

The data will be made available on request from the corresponding author.

## Appendix A

**Table A1:** Definition of the main variables.

Variable Type	Variable Name	Variable Symbol	Variable Definition
Explained Variable	Innovation investment	R&D	Natural logarithm of R&D expenses
	Innovation output	Patents	The number of patent applications, including the total number of applications for invention patents, designs and utility models
		LPatents	Natural logarithm of the number of patent applications
Explanatory Variable	Environmental uncertainty	LPatents	The industry-adjusted ratio of the standard deviation of sales revenue in the past 5 years to the average value of sales revenue in the past 5 years
Moderator	government subsidy	SUB	Government subsidy/total assets
	Information transparency	Trans	Accrued surplus calculated by the revised Jones model
Mediator	Second type of agency problem	Agency	Ratio of other receivables to total assets
Controlled Variable	Profitability	ROA	Net asset interest rate = Net profit/average total assets
	Company size	SIZE	Natural logarithm of total assets at the end of the year
	Investment opportunities	TOB	Tobin’s Q value
	Asset-liability ratio	LEV	Liabilities/assets of the balance sheet disclosed at the end of the year
	Two jobs in one	Dual	whether the general manager concurrently serve as the chairman of the board
	Industry	Ind	Ind is an industry dummy variable
	Year	Year	Year is the annual dummy variable

**Table A2:** Panel unit root test results.

Method	Statistic	Prob. **	Cross-Section	Obs
Levin, Lin and Chu t	−903.337	0.0000	1266	5951
Im, Pesaran and Shin W-stat	−60.8075	0.0000	921	4916
ADF-Fisher Chi-square	3506.88	0.0000	1266	5951
PP-Fisher Chi-	4333.32	0.0000	1266	5951

Note: ** Probabilities for Fisher tests are computed using an asymptotic Chi-square distribution. All other tests assume asymptotic normality.

**Table A3:** OLS Regression (panel data).

Variables	RD	LPatents
EU	−0.238 ***	−0.905 **
	(−3.48)	(−2.21)
ROA	0.482 ***	0.199
	(2.71)	(0.19)
SIZE	0.946 ***	0.659 ***
	(65.85)	(8.13)
LEV	−0.345 ***	2.521 ***
	(−3.09)	(3.82)
TOB	0.004	0.074 *
	(0.59)	(1.94)
JYYZ	0.000	−0.000
	(1.31)	(−0.39)
XJBL	−0.070 ***	−0.052
	(−4.35)	(−0.58)
Cash	0.321 ***	−0.160
	(2.75)	(−0.24)
Dud	−0.205	−0.938
	(−1.02)	(−0.79)
CGB	0.002	0.020 **
	(1.35)	(2.52)
Dual	−0.016	−0.098
	(−0.73)	(−0.78)
LDBL	0.005	0.100
	(0.48)	(1.50)
QYCS	−0.042 ***	−0.210 **
	(−3.03)	(−2.41)
Constant	−2.835 ***	−12.325 ***
	(−8.53)	(−6.62)
Observations	8,442	2,131
R-squared	0.434	0.077
Number of firms	1,879	891
Adjusted R^2^	0.270	−0.602
F	386.0	7.875

Note: *, ** and *** represent significant at the 10%, 5%, and 1% confidence levels, respectively. The t value is in parentheses.

**Table A4:** Poisson regression (panel data).

Variables	Patents
EU	−0.197 ***
	(−7.85)
ROA	2.959 ***
	(39.21)
SIZE	0.916 ***
	(281.78)
LEV	2.379 ***
	(49.34)
TOB	0.220 ***
	(75.99)
JYYZ	−0.004 ***
	(−37.31)
XJBL	−0.148 ***
	(−20.66)
Cash	2.198 ***
	(41.19)
Dud	1.872 ***
	(39.59)
CGB	0.015 ***
	(66.57)
Dual	−0.052 ***
	(−8.28)
LDBL	0.057 ***
	(15.24)
QYCS	−0.464 *** (−55.65)
Industry Year	Control Control
Constant	−19.266 ***
Pseudo R^2^	(−192.73) 0.4059

Note: *** represents significant at the 1% confidence level. The t value is in parentheses.
